# Mapping of possible prion protein self-interaction domains using peptide arrays

**DOI:** 10.1186/1471-2091-8-6

**Published:** 2007-04-12

**Authors:** Alan Rigter, Jan PM Langeveld, Drophatie Timmers-Parohi, Jorg G Jacobs, Peter LJM Moonen, Alex Bossers

**Affiliations:** 1 Central Institute for Animal Disease Control (CIDC-Lelystad), Wageningen UR Dept. of Bacteriology and TSEs, P.O. Box 2004, 8203 AA Lelystad, The Netherlands; 2 Pepscan Systems B.V., Edelhertweg 13, 8219 PH Lelystad, The Netherlands

## Abstract

**Background:**

The common event in transmissible spongiform encephalopathies (TSEs) or prion diseases is the conversion of host-encoded protease sensitive cellular prion protein (PrP^C^) into strain dependent isoforms of scrapie associated protease resistant isoform (PrP^Sc^) of prion protein (PrP). These processes are determined by similarities as well as strain dependent variations in the PrP structure. Selective self-interaction between PrP molecules is the most probable basis for initiation of these processes, potentially influenced by chaperone molecules, however the mechanisms behind these processes are far from understood. We previously determined that polymorphisms do not affect initial PrP^C ^to PrP^Sc ^binding but rather modulate a subsequent step in the conversion process. Determining possible sites of self-interaction could elucidate which amino acid(s) or amino acid sequences contribute to binding and further conversion into other isoforms. To this end, ovine – and bovine PrP peptide-arrays consisting of 15-mer overlapping peptides were probed with recombinant sheep PrP^C ^fused to maltose binding protein (MBP-PrP).

**Results:**

The peptide-arrays revealed two distinct high binding areas as well as some regions of lower affinity in PrP^C ^resulting in total in 7 distinct amino acid sequences (AAs). The first high binding area comprises sheep-PrP peptides 43–102 (AA 43–116), including the N-terminal octarepeats. The second high binding area of sheep-PrP peptides 134–177 (AA 134–191), encompasses most of the scrapie susceptibility-associated polymorphisms in sheep. This concurs with previous studies showing that scrapie associated-polymorphisms do not modulate the initial binding of PrP^C ^to PrP^Sc^. Comparison of ovine – and bovine peptide-array binding patterns revealed that amino acid specific differences can influence the MBP-PrP binding pattern. PrP-specific antibodies were capable to completely block interaction between the peptide-array and MBP-PrP. MBP-PrP was also capable to specifically bind to PrP in a Western blot approach. The octarepeat region of PrP seems primarily important for this interaction because proteinase K pre-treatment of PrP^Sc ^completely abolished binding.

**Conclusion:**

Binding of MBP-PrP to PrP-specific sequences indicate that several specific self-interactions between individual PrP molecules can occur and suggest that an array of interactions between PrP^C^-PrP^C ^as well as PrP^C^-PrP^Sc ^may be possible, which ultimately lead to variations in species barrier and strain differences.

## Background

Transmissible spongiform encephalopathies (TSEs) are fatal neurodegenerative disorders characterized by formation and accumulation of partially protease resistant prion protein (PrP^Sc^) mainly in tissues of the central nervous system. TSEs (or prion diseases) include (among others) familial, sporadic and variant Creutzfeldt-Jacob disease in humans, bovine spongiform encephalopathy (BSE) in cattle, and scrapie in sheep. Formation of PrP^Sc ^is a posttranslational process and involves refolding (conversion) of the host-encoded prion protein (PrP^C^) into partially protease resistant forms (PrP^Sc^) [1]. Since no other proteins are known to be involved in this conversion, the existence of a specific and probably efficient self interaction between PrP molecules must be considered.

The molecular mechanism involved in PrP conversion is not well understood, but polymorphisms in PrP have been shown to be of importance in both interspecies and intraspecies transmissibilities [2] and cell-free conversion of PrP^C ^provides an excellent *in vitro *model in which relative amounts of produced proteinase K (PK) resistant PrP reflect important biological aspects of TSEs at the molecular level [2-9]. Whereas differences in susceptibility of- and transmissibility in sheep can largely be explained at the molecular level by the effects of single polymorphisms in PrP^C ^or PrP^Sc ^on PrP conversion [6,10-12], the exact molecular mechanism of disease development modulation by polymorphisms is still unknown, however we previously showed that disease associated polymorphisms do not affect the initial binding of PrP^C ^to PrP^Sc ^[13]. Hölscher *et al *showed by deletion of residues 114–121 (mouse PrP) the necessity of the highly amyloidogenic AGAAAAGA motif in conversion of PrP^C ^to PrP^Sc ^[14]. Many other studies have revealed the importance of the PrP regions encompassing amino acid sequence (AA) 90–120 (which confirms the importance of AGAAAAGA) [15-17] and 132–156 [8,15,18-27]. However, to our knowledge no attempts have been made to systematically map all possible AA involved in PrP interaction (During review of this manuscript a study with complementary results directed at the identification of regions of PrP^C ^that tightly bind to PrP^Sc ^by using a limited set of sequential 24-mer polypeptides motif grafted onto an antibody was published [28]. Our study has its focus however, on systematical domain mapping at the single amino acid level by using a complete set of overlapping 15-mer PrP derived peptides). In order to elucidate which AA of PrP capable of interaction are involved in the primary interaction of PrP^C ^to PrP^Sc^, a peptide-array based on linear PrP sequences comprising the complete PrP sequence was utilized to determine which residues of PrP are capable of interacting with PrP^C^.

## Results

### MBP-PrP expression and analysis

**Figure 1:**
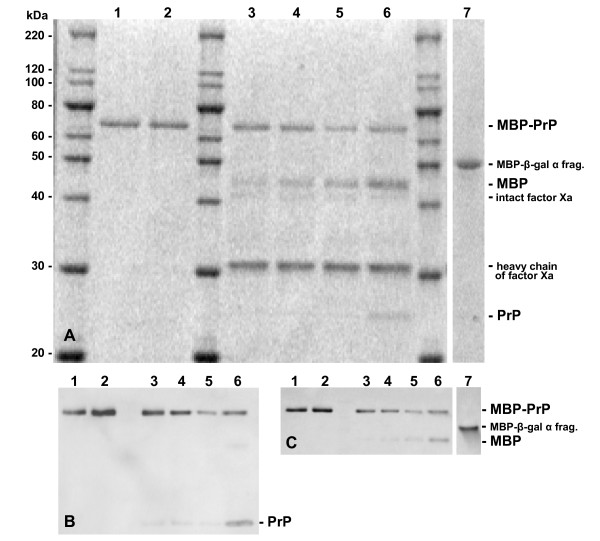
**Analysis of MBP-PrP and MBP expression and MBP-PrP digestion by Factor Xa**. Lane 1 contains untreated MBP-PrP, whereas lane 2 contains a mock digestion of MBP-PrP. MBP-PrP was digested with 1% w/w factor Xa and during digestion samples were taken at 2, 4, 7 and 24 hours (lanes 3,4,5 and 6 respectively). All samples were run on SDS-PAGE and the gel was stained with Sypro Orange (total protein stain, panel A) before western blotting and subsequent immunodetection using either a PrP-specific monoclonal antibody (9A2, panel B) or MBP-specific monoclonal antibody (α-MBP, panel C). Expression of MBP, expressed from the pMAL-c2X vector with no insert (MBP-β-gal α fragment), was analyzed by Western Blot using either 9A2 or α-MBP (lane 7, panel A & C respectively)

Expression of maltose binding protein N-terminally fused to PrP (MBP-PrP) revealed a mainly soluble recombinant MBP-PrP of approximately 70 kDa (Fig. 1, lanes 1 & 2) and is readily detected in Western blot using a PrP-specific antibody (9A2, Fig. 1B) or a MBP specific antibody (Fig. 1C). The MBP-PrP fusion-protein could be purified using the amylase-resin column and the naked PrP protein could be obtained by digestion with protease Factor Xa, indicating accessible folding (Fig. 1, lanes 3–6). After 24 hours approximately 45% of MBP-PrP was digested by factor Xa, however when aided by addition of 0.01% SDS factor Xa completely digested MBP-PrP within 24 hours (data not shown). Monoclonal (9A2 and 94B4, Table 1) and polyclonal (R521, epitope AA100–102 [29,30]) PrP-specific antibodies (Table 1) with specificity for epitopes dispersed throughout the PrP-protein detected MBP-PrP in Western blot. MBP expressed without additional fusion protein (PrP), which frequently served as negative control in this study, was also of homogeneous quality (Fig 1, lane 7) and of expected size (MBP-β-gal α fragment, 50.8 kDa) which is somewhat larger (as expected) than MBP cleaved from the fusion protein after factor Xa digestion (42.5 kDa). Though we did not study its physical state, the soluble MBP-PrP product used is most likely in a monomeric or low oligomeric state representative for PrP^C^, with a secondary structure that is high in alpha helix and random coil and low in beta-sheet [31,32].

**Table 1:** Monoclonal antibody overview

**antibody**	**epitope^a^**	**position^b^**	**blocking^C ^(μg/ml)**	**reference^d^**
**100B3**	KRPKP	26–30	~0,5	Thuring, 2005 [30]
**SAF32**	QPHGGGW ^e^	54–92	25,0	Feraudet, 2005 [51]
**9A2**	WNK	102–104	4,0	Langeveld, 2006 [47]
**6C2**	HVAGAAA	114–120	20,0	this paper
**6H4**	DYEDRYYRE	147–155	1,0	Korth, 1997 [18]
**94B4**	HTVTTTTK	190–197	10,0	Thuring, 2004 [52]
**M7**	QQSYGQEP	n.a.	n.a.	Bakker, pers. comm.

^a ^amino acid sequence of the reported antibody epitope using peptide mapping techniques

^b ^position of the antibody epitope(s) in the ovine protein sequence

^c ^concentration at which the antibody completely blocked binding on the peptide-array

^d ^publication in which the epitope mapping results are reported

^e ^epitope of octarepeat (partially); occurs as 5 respectively 6 successive sequences in ovine and bovine PrP

### Binding domains of ovine PrP

**Figure 2:**
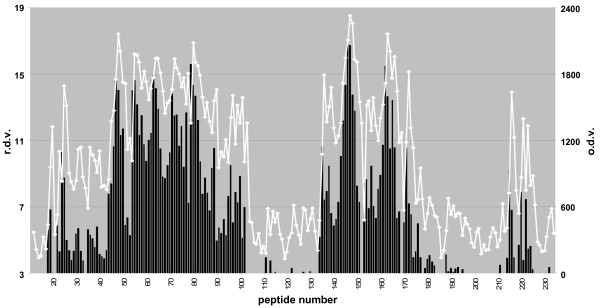
**Peptide-array binding pattern of MBP-PrP**. Dual plot of MBP-PrP binding to the ovine PrP peptide-array. The relative density value (r.d.v.) was calculated by dividing the optical density value (o.d.v.) by the background and binding was considered relevant when at least 3 consecutive peptides showed binding values of at least 3 times the background. The unprocessed optical density values (left X-axis) of each peptide (Y-axis, peptide number) are plotted in the graph, while relative density values (right X-axis) of each peptide are plotted in the column graph.

**Figure 3:**
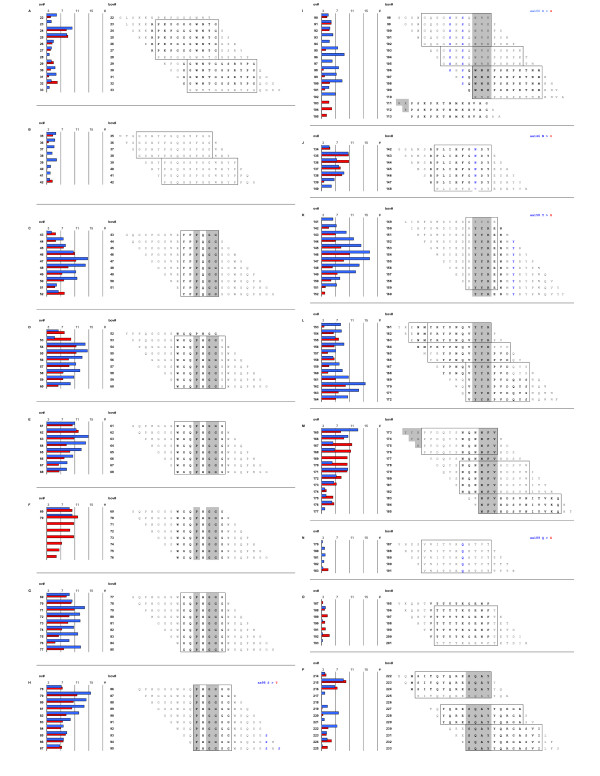
**Correlation of relative binding pattern, peptide amino acid sequence and species variation between ovine and bovine PrP**. Horizontal bars represent r.d.v of each peptide in a binding region with the position of each peptide indicated for ovine (left numbers) and bovine (right numbers) peptide array. Amino acid sequences are the ovine peptide sequences of all peptides in designated binding regions. The consensus domains found within each binding region for the ovine sequence are boxed and the consensus domains of the corresponding peaks found with the bovine peptide-array are in bold font. Blue bars represent r.d.v of ovine peptide-array, red bars r.d.v. of bovine peptide-array. Substitutions in bovine PrP are mentioned at right top side of each panel (panels H, I, J, K, N).

Using solid-phase arrays of 15-mer overlapping peptides systematically covering the whole mature part of PrP^C^, MBP-PrP was allowed to bind with the peptide-array, with the prospect that this would yield information on interaction sites between its PrP moiety and the linear peptides. Indeed interaction between the individual PrP sequences (peptides) and MBP-PrP was sufficient for immunodetection, resulting in a reproducible binding pattern (Fig. 2, line graph). This binding pattern, expressed in relative density values (Fig. 2, column graph), was characterized by two distinct high binding areas (peptides 43–102 and 134-177 respectively) as well as some lower binding areas. Analysis of the correlating peptide sequences revealed that these areas usually were characterized by consensus sequences which suggested the existence of the following interaction domains for the mature part of PrP^C ^(Fig 3). Two consecutive binding peaks with peptides 22–28 + 29–33 (Fig. 3A) have [33-**GWNTG**-37] (ovine protein sequence position used throughout) as their consensus domain, followed by two consecutive minor binding peaks with peptides 35–38 + 39–42 (Fig. 3B) with [42-**PGQGSPGG**-49] as the common domain. The first high binding area is comprised of peptides 43–102, and encloses only two likely consensus domains: on the one hand peaks 43–52 (Fig. 3C), 53–60 (Fig. 3D), 61–68 (Fig. 3E), 69–77 (Fig. 3G) and 78–87 (Fig. 3H) each recognized an octarepeat with [**PxGG**, x = Q or H] as the consensus domain and on the other hand peaks 90–93, 94–97, 98–102 (Fig. 3I) with [102-**WNK**-104] as a common domain. The second high binding area is comprised of peptides 134–177 and encloses three likely consensus sequences. The shared sequence for peptides 134–136, 137–140 (Fig. 3J) is [140-**PLIHFGNDY**-148], for peptides 141–151 (Fig. 3K, AA152–154) and 153–155, 156–158, 59–164 (Fig. 3L, AA165–167) is [**YYR**] and for peptides 165–168, 170–173, 174–177 (Fig. 3M) is [177-**NFV**-179] respectively. The remaining lower binding areas also enclose three likely binding domain consensuses; [183-**VNITVKQHTVT**-193] for peptides 179–183 (Fig. 3N), [192-**TTTTKGENFT**-202] for peptides 188–193 (Fig. 3O) and [225-**SQAY-**228] for peptides 214–217, 219–221, 222–225 (Fig. 3P).

### Ovine versus bovine peptide-array

To further assess the extent of the specificity of the binding pattern found, MBP-PrP was also tested against a bovine PrP peptide-array. This yielded a rather similar binding pattern compared to the results with ovine PrP peptide array but with some differences. The binding pattern on the bovine peptide-array (Fig. 3, red bars) was compared to that on the ovine peptide-array (Fig. 3, blue bars). As expected an extra octarepeat was found (Fig. 3F), and of the six amino acid differences between the ovine – and bovine peptide arrays only two resulted in a difference in binding. Binding with the peptides containing the ovine to bovine substitutions S98A (ovine numbering used throughout, Fig. 3H–I), S146A (Fig. 3J) and Y158H (Fig. 3K) were comparable on both the ovine and bovine peptide-array, whereas the S100G (Fig. 3I) and Q189E (Fig. 3N) did result in altered binding patterns. No binding was found with peptides containing the I208M substitution (data not shown). Some differences in binding without a direct apparent reason were observed. Differences in the relative level of binding was observed with peptides 165–177 (Fig. 3M, bo# 173–785), 187–193 (Fig. 3O, bo# 195–201) and 220–222 (Fig. 3P, bo# 227–229), but these differences did not translate in differences in the determined consensus domains. However, binding with the array of bovine peptides 35–42 (Fig. 3B) remained below the cutoff value (3 times background), whereas low binding with peptides 103–105 (Fig. 3I) was observed with the bovine peptide-array but not with the ovine peptide-array.

### Peptide-array controls

**Figure 4:**
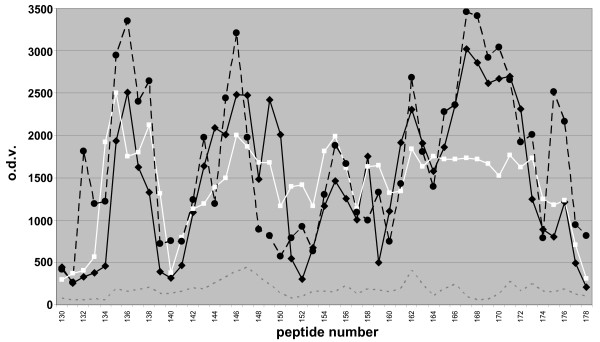
**Peptide array controls**. Optical density value plot of MBP-PrP isolate 1 measured on minicard 1 (black line), MBP-PrP isolate 2 on minicard 1 (black dotted line), and MBP-PrP isolate 1 on minicard 2 (white line) and of MBP (background, grey dotted line) on peptide-array. For differences between isolates, compare black and white line. For differences between peptide synthesis batches compare black and black dotted line.

Several control tests were carried out to determine the viability of the peptide-array to obtain PrP-specific binding patterns. Only minor non-significant differences in binding pattern were seen as a result of varying concentration of MBP-PrP (except for the expected difference in optical density value [o.d.v.]), storage buffers for MBP-PrP, peptide synthesis methods, or peptide-array batches (Fig. 4). Also, no significant binding was observed with each separate antibody or in the combination used for detection (thus in the absence of MBP-PrP). Furthermore no binding of MBP with the PrP peptide-array was observed. Interestingly the o.d.v. decreased after prolonged storage of MBP-PrP in PBS + 0.1% SB3–14. Further examination of the isolate showed that MBP-PrP had precipitated, indicating that interaction between the peptides and MBP-PrP only occurs when the latter is soluble. Furthermore, MBP-PrP was tested on an unrelated peptide-array containing overlapping peptides covering the sequence of VP2 of canine parvovirus yielding not any significant binding domains. All these controls confirm that binding of MBP-PrP to the PrP-peptides was as a result of the PrP moiety of MBP-PrP, and that this binding was PrP-specific.

### Antibody blocking of peptide-array binding pattern

**Figure 5:**
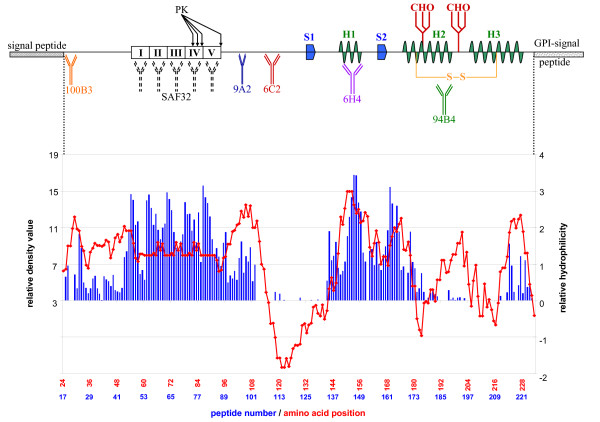
**Overview of PrP^C ^secondary structures and antibody epitopes versus peptide-array binding pattern and Kyte-Doolittle hydrophilicity plot**. Schematic representation of PrP^C ^showing signal sequences, β-sheets (S1, S2), α-helices (H1, H2, H3), disulfide bridge site (S-S), glycosylation sites (CHO) and relative positions of the antibodies used in this study. The sequence of PrP is lined up with both the Kyte-Doolittle hydrophilicity plot (negative = hydrophobic and positive = hydrophilic) and the relative binding pattern found with the ovine peptide-array.

To find a correlation with structural properties, the relative binding pattern of MBP-PrP on the peptide array was compared to the Kyte-Doolittle hydrophilicity plot of mature PrP^C ^revealing a high correlation between hydrophilic (exposed) regions of PrP^C ^and binding pattern regions (Fig. 5). Even though the correlation was not absolute, it was necessary to determine if the binding pattern could be blocked with PrP-specific antibodies. Therefore several monoclonal antibodies with epitopes at different sites throughout PrP^C ^(Table 1, Fig. 5) were tested. Pre-incubation (1 hour at room temperature) of MBP-PrP with all the tested PrP-specific monoclonal antibodies resulted in a concentration-dependant blocking of MBP-PrP binding over the whole set of PrP-peptides, albeit at different antibody concentrations (Table 1). No blocking of the MBP-PrP binding pattern occurred after pre-incubation with the unrelated Mycobacterium specific antibody M7. To ensure that blocking of the binding pattern is a result of immune-complex formation between antibody and MBP-PrP and not of incidental aspecific aggregation of MBP-PrP, a Mab-aggregation test for each PrP-specific antibody was carried out. Comparative amounts of MBP-PrP and antibody (necessary for blocking) were incubated. The soluble and insoluble fraction were separated by centrifugation at 20.000 × g and analyzed on SDS-PAGE, resulting in 75 ± 16% of MBP-PrP and 85 ± 7% of antibody detected in the supernatant fraction (data not shown), indicating that if an immune-complex is formed this complex remains soluble. Therefore formation of a soluble immune-complex must be responsible for loss of binding in the peptide-array instead of diminished binding as a result of aspecific aggregation. In addition preliminary results indicate that some selected peptides are also capable of blocking MBP-PrP binding to the peptide-array, confirming that binding of MBP-PrP to the PrP-peptides was as a result of the PrP moiety of MBP-PrP and PrP-specific.

### MBP-PrP mediated detection (reverse detection) of PrP in Western blot

**Figure 6:**
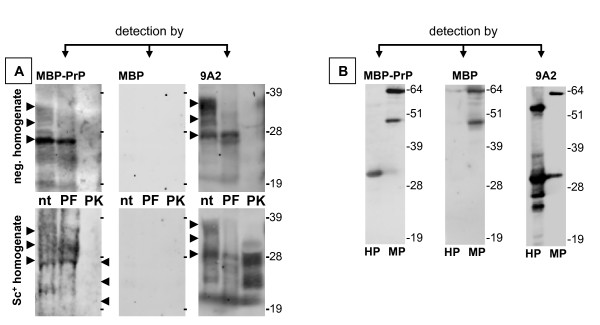
**Interaction of recombinant MBP-PrP with various species of PrP in Western blot**. Samples containing either brain derived PrP [A] or recombinant derived PrP [B] were analyzed by SDS-PAGE and subsequent Western blotting. PrP was detected using MBP-PrP (left panels), MBP alone (center panels) or PrP-specific antibody 9A2 (right panels). Refer to the Methods section on "reverse detection assay" for specific details concerning immunodetection of membrane bound PrP. Aliquots from PrP^Sc ^negative ([A] upper panels) and PrP^Sc ^positive brain ([A] bottom panels) homogenates were either treated with PNGase F (PF) or proteinase K (PK) and compared to non-treated (nt) aliquots of brain homogenate. His-PrP ([B], lanes marked HP) was used as a positive PrP-control and MBP-PrP ([B] lanes marked MP) was used as a positive detection control. Arrow heads indicate the approximate position of the un-glycosylated, mono- and di-glycosylated PrP isoforms.

To further confirm the specificity of the observed PrP-PrP interaction, MBP-PrP was used as a detector in Western blot to further study its affinity towards intact PrP^C ^in a brain homogenate. MBP-PrP could be used to detect recombinant His-tagged PrP (Fig. 6B, left panel lane marked HP) and intact PrP in both scrapie positive and negative brain homogenates (Fig. 6A, left panel lanes marked nt), albeit with a lower sensitivity under the standard Western blot conditions using monoclonal antibody 9A2 (Fig. 6A, compare left and right panel). MBP-PrP seems to preferably detect the un-glycosylated PrP in the scrapie negative brain homogenate (in contrast to the scrapie positive homogenate). Correspondingly PNGase F treatment of the brain homogenates did not alter the capability of MBP-PrP to detect PrP in brain homogenates (Fig. 6A, right panel lanes marked PF) even though detection with 9A2 showed decreased levels of glycosylated PrP (Fig. 6A, left panel lanes marked PF). MBP-PrP detection of PrP in the PNGase F treated scrapie positive homogenate still shows some detection of the different glycosylation forms (Fig. 6A, right lower panel lane marked PF). Comparison of MBP-PrP detection of PrP in the PNGase F treated – and non-treated scrapie positive brain homogenate samples shows that after PNGase F treatment the amount of di-glycosylated PrP has decreased while mono-glycosylated PrP has increased. Therefore the detection of PrP glycoforms after PNGase treatment is not due to aspecific binding of MBP-PrP, but rather a result of incomplete de-glycosylation of PrP in this particular sample. In contrast, PK treatment of the brain homogenate abrogated MBP-PrP detection (Fig. 6A, lanes marked PK) whereas immunodetection using monoclonal antibody 9A2 clearly shows the presence of PK-resistant PrP^Sc ^(Fig. 6A, right panel lane marked PK) in the scrapie positive brain homogenate. As a control the same samples were tested in Western blot using free MBP, resulting in no detectable signal with either His-PrP or any of the brain homogenate samples (Fig. 6, center panels). Only MBP-PrP (or MBP) was detected (Fig. 6B, center panel, positive detection control), thus proving that the detection with MBP-PrP was PrP-specific.

## Discussion

### Binding domains of ovine PrP^C^

Probing for possible PrP interaction domains using MBP-PrP and a solid-phase PrP peptide-array resulted in PrP specific interaction between specific PrP-sequences (peptides) and MBP-PrP. This probing revealed several likely interaction domains encompassed in two distinct high binding areas and some lower binding areas which will be discussed below in relation to structural features of PrP^C^. Suggested properties in conversion, the species barrier and self-interaction sites as hypothesized in structural models will be discussed.

The first distinct high binding area contains two different interaction domain consensuses. The domain [**PHGG**] is repeated five times in the ovine peptide-array and six times in the bovine peptide-array (Fig. 3C–H) and is part of the octarepeat sequence PHGGGWGQ, except for the first octarepeat (Fig. 3C) where H is replaced with a Q. This substitution is considered neutral [33], confirmed by the lack of effect on the binding pattern found, even though Q is a polar residue and the weak positive charge of H is neutralized. The octarepeats are an epitope for antibodies inhibiting PrP^Sc ^propagation in cell culture [26]. This study further reveals domain [102-**WNK**-104] (Fig. 3I) as a domain involved in PrP-PrP interaction. These three AAs are part of the epitope of the motif-grafted antibody containing mouse AA 89–112, which is capable of selective immuno-precipitation of infectivity [34].

The second high binding area contains 3 different interaction domain consensuses. This area also includes most of the polymorphisms found in sheep PrP^C^. The domain [140-**PLIHFGNDY**-148] (Fig. 3J) is situated between the first β-sheet (Fig. 5, S1) and first α-helix (Fig. 4, H1; the D and the Y are actually the first AA in α-helix 1) in PrP^C ^and is part of the epitope of the motif-grafted antibody containing mouse AA 136–158 (together with [152-**YYR**-154]), capable of selective immuno-precipitation of prion infectivity [34]. This domain encompasses the two amino acid positions which appear to fully control species-specific kinetics of PrP23–144 [35] by affecting amyloid fibril conformation, thus limiting which PrP^C ^molecule can adapt to the conversion seed. Therefore this domain is most likely involved in the species barrier and/or indirectly determines the susceptibility of sheep PrP to scrapie, maybe by influencing the accessibility of this domain and thus the adaptability of PrP^C ^to the conversion seed. Involvement of this region in adaptability between species was also concluded for human, mouse and hamster prions [35]. The [**YYR**] sequence occurs twice in the PrP sequence. AA [152-**YYR**-154] (Fig. 3K) is situated within the first α-helix (Fig. 5, H1) and AA [165-**YYR**-167] (Fig. 3L) is situated within the second β-sheet (Fig. 5, S2) of PrP^C^. The charged residues in the first α-helix, especially at residues 151, 152 and 154 (D, Y and R respectively) have also been shown to be a determinant of conversion [36]; substitutions of neutral amino acids or oppositely charged residues impaired conversion. Furthermore, [152-**YYR**-154] (together with [140-**PLIHFGNDY**-148]) is part of the of the epitope of the motif-grafted antibody containing mouse AA 136–158, capable of selective immuno-precipitation of prion infectivity [34]. The [**YYR**] domain has also been described as the epitope of an antibody that selectively recognizes PrP^Sc ^[37], and is part of several antibody (fragment) epitopes that prevent scrapie infection in tissue culture [18,19,23,25,26] or in a mouse model [24]. The putative domain [177-**NFV**-179] is the common AA sequence of three consecutive peaks of which the fist two peaks encompass amino acids of a peptide (corresponding to AA 163–176) that inhibits in vitro conversion [15]. However, because of the third peak it is more likely that this domain is involved in self-interaction.

The remaining lower binding areas are more difficult to interpret. The domain consensus for the peptides 22–33 (Fig. 3A) seems to be [33-**GWNTG**-37], but may be a result of a cross-interaction with WN, instead of binding to domain [102-**WNK**-104] within the first high binding area. Binding with domain consensus [42-**PGQGSPGG**-49] (Fig. 3B) is relatively low and may likely be due to cross-interaction with the proline (P) and two consecutive glycines (G) of these peptides, instead of binding to the consensus octarepeat domain [**PxGG**]. Binding with the domains [183-**VNITVKQHTVT**-193] (Fig. 3N) and [192-**TTTTKGENFT**-202] (Fig. 3O) is also (very) low and these domains comprise the second helix in PrP^C ^(Fig. 5, H2) which in turn may explain the relative low binding due to the structured nature of this part of the protein. It is not clear what the importance is of these domains, but part of the latter domain is contained within a peptide (corresponding to AA 197–220) that inhibits in vitro conversion [15] as well. The last low binding domain found is [225-**SQAY**-228], which (together with [**YYR**]) is part of the non-linear epitope of the PrP^Sc ^specific monoclonal antibody 15B3 [18].

Several studies identified antibodies [18,19,23-26] or peptides [15] able to inhibit prion propagation. The binding domains found with the peptide-array containing AA corresponding with these antibody epitopes or peptides may also be (in)directly involved in conversion. However, the inhibitory effects of antibodies seems simply due to steric hindrance preventing any PrP interaction at all (also confirmed by our peptide-array blocking results), making the binding domains corresponding to the conversion inhibiting peptides (Fig. 3M, peak1+2 and Fig. 3O) the more likely candidate domains influencing conversion.

Our data are in line with a theoretical two rung β-helical model described by Langedijk *et al. *[38], which tries to explain how AA sequence and secondary structure could explain strain properties and the species barrier. The binding domains found by peptide-array are all exposed in the periphery of the proposed fibril. The first distinct high binding domain makes up the incoming protein chain, whereas the second distinct high binding area forms the loop connecting the two-rung β-helical core with the α-helices (the outgoing protein chain). Furthermore, no binding is observed between the two distinct high binding areas and the AA sequence of these peptides correlate with the predicted two rung β-helical core.

Our data provide insight in the possible interaction domains of PrP^C ^with itself or PrP^Sc^, and most of the domains identified are likely to be involved in PrP^C ^self-interaction. This may involve dimerisation of PrP^C ^and/or formation of a trimer of three PrP^C ^molecules as suggested in the theoretical two-rung β-helical model for PrP stacking during PrP^Sc ^induced fibrillization [38]. If one of the identified high binding domains is of influence on conversion, this possibly is exerted during pre-oligomerisation, which is an inefficient process. On the other hand, a direct effect on PrP^C^-PrP^Sc ^binding can not be excluded.

### Ovine versus bovine peptide-array

In addition to the ovine peptide-array, a similar array of overlapping 15-mer peptides bovine peptides was used. Since there are several sequential differences between bovine and ovine PrP this may be of influence on the overall binding pattern found with MBP-PrP (ovine PrP). Generally it seems that binding of MBP-PrP is somewhat less efficient (strong) with the bovine peptide-array compared to binding with the ovine peptide-array. However, this had no significant effects on the relative binding pattern. In the amino acid sequence of bovine PrP there is an extra octarepeat as well as six amino-acid substitutions. Only the largest differences will be discussed here. The non-discussed differences may well be the result of minor methodological variations when producing the peptide-arrays.

As expected, an extra octarepeat (Fig. 3F) was evident that confirmed the octarepeats consensus binding domain [PHGG]. Only two out of six amino acid substitutions were of influence on the binding pattern. At first glance the amino acid substitutions at AA 98 and 100 (sheep numbering used throughout) are seemingly both of influence on binding with peptides 93–97 (Fig. 3I, bo# 98–113), allowing detection of the consensus binding domain [102-**WNK**-104] only when these AA were no longer present in the peptides. The supposedly neutral substitution of glycine for serine at AA 100 is most likely responsible for the observed differences in binding and may be attributed to the greater conformational flexibility of G, affecting availability of other AA's for interaction with MBP-PrP. Taking this in account, binding with peptides 90–92 should be a result of cross-binding with two consecutive Glycines present in these peptides in stead of binding with the consensus octarepeat domain. Glutamatic acid only contains an oxygen in place of the amido group in glutamine and therefore these AA's are considered readily interchangeable [33]. However we observed that the substitution of glutamatic acid for glutamine at AA 189 did affect binding (Fig. 3N) on the bovine peptide-array with peptides 179–183; Both AA's are polar, but where glutamine interacts with other polar or charged atoms with its polar side chains, glutamatic acid is negatively charged and is frequently involved in salt-bridges and/or glutamatic acid interacts with positive charged AA's to form hydrogen bonds. These differences in AA reactivity are most probably responsible for the observed difference in binding between ovine and bovine PrP peptide sequences.

Analysis and comparison of the relative (consensus) ovine – and bovine peptide-array revealed that the detection of potential PrP-PrP interaction domains using this method is robust as well as sensitive to differences in structural flexibility and/or amino acid differences. Therefore, this peptide-array approach provides a possibly valuable tool to assess the influence of disease associated polymorphisms on available interaction domains and to test for PrP-PrP binding inhibitors potentially useful in therapy (i.e. antibodies or peptides).

### Antibody blocking of peptide-array binding pattern

All monoclonal antibodies (mab) recognizing PrP (Table 1, Fig. 5) are capable of blocking the complete binding pattern. Differences in the antibody concentration necessary for complete blocking are likely due to epitope availability and/or affinity for PrP. Complete blocking of the binding pattern can best be explained by steric hindrance of the antibodies preventing any interaction. It has been described that binding of a monoclonal antibody at the N-terminus of human PrP influences epitope availability at the C-terminus [39] and similar events may also attribute to completely abolishing the binding pattern. Furthermore, structural studies of PrP^Sc ^that resulted in prion propagation/fibrillization models [38,40,41], suggest that PrP-PrP interaction depends on the structure of the whole protein (not just the trimer or dimer core). These findings corroborate the notion that antibodies inhibiting prion propagation probably do so by preventing the interaction between PrP^C ^and PrP^Sc ^or between PrP^C ^molecules themselves in the pre-oligomerisation phase.

### MBP-PrP mediated detection (reverse detection) of PrP in Western blot

By using MBP-PrP as the detecting agent in Western blot we showed that binding of MBP-PrP to the peptide-arrays is PrP specific and indicative of a PrP-PrP interaction. MBP-PrP seems to preferably detect un-glycosylated PrP^C ^in the scrapie negative brain homogenate. The exact reason for this preference is unclear, but does not seem to be due to the lack of glycosylated isoforms in the brain homogenate (except after de-glycosylation with PNGase F) as shown by immuno-detection with 9A2. It may be possible that the determined interaction domains do not (only) interact with the same amino acid motif (self-self), but that an intramolecular cross-interaction between different domains can also occur. The peptide-array data has revealed two high binding areas, one of which contains the N-terminal octarepeats and the other contains the first α-helix, second β-sheet up to the second α-helix directly adjacent to the glycosylation sites (Fig. 5). When PrP^C ^in the homogenate is glycosylated this may sterically hinder binding of MBP-PrP to PrP^C^. Glycosylation is suggested to have a role in prion strain maintenance and the species barrier [42] by modulating the fidelity of interaction, which may explain the favorable binding of un-glycosylated PrP by MBP-PrP (which is also un-glycosylated, indicating that binding preferably occurs between compatible glycosylated molecules) in the scrapie negative homogenate. However, in the scrapie positive brain homogenate detection of PrP by MBP-PrP is more diffuse and might suggest that MBP-PrP detection of PrP in Western blot is due to interaction with both full-length PrP^C ^as well as full length denatured PrP^Sc^, which is comparable to PrP^C^. Alternatively, PrP^Sc ^might be partly endogenously truncated resulting in more heterogeneous binding of MBP-PrP to all glycosylation forms. In contrast, MBP-PrP detects un-glycosylated PrP as well as both mono – and di-glycosylated PrP in the scrapie positive homogenate, even though un-glycosylated PrP usually is the lesser component in the PrP-triplet of scrapie sheep brain samples, which may be indicative for preferable binding of un-glycosylated PrP in the scrapie positive homogenate. However interpretation of these results is difficult and in order to elucidate the precise effects of glycosylation on binding between PrP molecules, interaction should be studied under more native conditions. When proteinase K treatment was applied MBP-PrP did not detect PK-resistant PrP^Sc^. This indicates that in order for MBP-PrP to detect PrP in brain homogenates (under the conditions used) full length (at least containing the high binding area with octarepeats) PrP molecules are required. It may be hypothesized that the first high binding area containing the octarepeats aids in stabilization of PrP self-interaction, perhaps by intramolecular interaction with other mapped interaction domains. This extra stabilization in turn allows further immunodetection in Western blot (under the conditions used). These results combined with the results obtained by peptide-array analysis support the concept of self-interactive domains of PrP^C^.

## Conclusion

In summary, probing for possible interaction domains in PrP using a solid phase PrP peptide-array revealed that specific interactions take place between individual PrP molecules. Ten possible consensus binding domains were found, which includes one domain that likely is due to a cross-reaction with the octarepeat domain consensus – and for two domains it remains unclear what their importance is. The remaining seven domains are most likely involved in PrP^C ^self-interaction. Furthermore, MBP-PrP was also capable to specifically bind to full length PrP^C ^and PrP^Sc ^bound PrP^C ^in Western blot confirming PrP-PrP specific interaction. Together these results indicate that in addition to direct PrP^C^-PrP^Sc ^interactions several other molecular interactions between PrP^C ^molecules/sequences themselves may also be possible, facilitating initial steps in the oligomerisation process.

The PrP peptide-array may additionally facilitate in gaining insight into effects of disease associated polymorphisms in PrP on PrP-PrP binding, and the subsequent molecular conversion of PrP^C ^into PrP^Sc^. The (self-)interaction domains described here may ultimately prove useful in the design of therapeutics interfering in the PrP-PrP binding process.

## Methods

### MBP-PrP construct

In order to obtain the PrP gene suitable for cloning into the pMAL Protein Fusion and Purification System (New England Biolabs), the mature part of the sheep PrP (ARQ) open reading frame (ORF) was PCR amplified using primers ShBo-F-DraI (GGTGGTTTTAAAAAGCGACCAAAACCTGG) and Sh-R-STOP (GGTGGTCTATGCCCCCCTTTGGTAATAAGCC). The resulting PrP (AA25–233), without its N -and C-terminal signal sequences, was cloned into a general TA-cloning vector (Invitrogen) and sequenced to exclude PCR artifacts. The PrP fragment was subsequently sub cloned using *Dra*I and *Eco*RI into the pMAL-c2X expression vector, resulting in the maltose binding protein (MBP) fusion to the N-terminus of PrP (MBP-PrP).

### MBP-PrP expression and purification

Expression and purification by affinity chromatography was performed as described in the manual of the pMAL Protein Fusion and Purifications System (method I; New England Biolabs) To improve binding of MBP-PrP to prevent formation of interchain disulfide upon lysis (as suggested in the protocol), β-mercaptoethanol was added. Quantity and quality of the eluted MBP-PrP was determined by SDS-PAGE (12% NuPAGE, Invitrogen). After separation the gel was either stained with Sypro Orange (total protein stain, Molecular probes) or analyzed by Western blotting and immunodetection of MBP-PrP with polyclonal antiserum R521-7 specific for PrP. To obtain MBP for cross-reaction aspecificity tests, the pMAL-c2x expression vector without insert was expressed and purified as described above.

### Peptide-array analysis

Synthesis of complete sets of overlapping 15-mer peptides were carried out on grafted plastic surfaces, covering the ovine or bovine PrP amino acid sequence of mature PrP (residues 25–234 of ovine and 25–242 of bovine PrP) [43]. The plastic surface consisted of 455, 3 μl wells on a credit-card size plastic (minicard) carrier. Peptide-arrays covering the ovine or bovine PrP amino acid sequence of mature PrP were custom synthesized through two different synthesis techniques: either all peptides of the array were synthesized in situ to the grafted plastic surface by step-by-step amino acid coupling or the peptides were synthesized separately and coupled as complete 15-mer peptides to each well at their C-terminus [44-46]. In subsequent ELISA analyses on the minicards, MBP-PrP was incubated as an antigen followed by immuno-screening with mouse anti-MBP monoclonal antibody (Mab) obtained from New England Biolabs and rabbit anti-mouse-IgG-peroxidase, or a rabbit anti-MBP Mab and swine anti-rabbit-IgG-peroxidase (from DAKO, Denmark). Blocking studies were performed by pre-incubating the MBP-PrP with a PrP-specific Mab before incubating the mixture as the antigen on the minicard. The background was determined by calculating the mean value of 20 peptides with low density values of which at least 5 peptides were in consecutive order. The relative density value (r.d.v.) was calculated by dividing the optical density value (o.d.v.) by the background and binding was considered relevant when at least 3 consecutive peptides showed binding values of at least 3 times the background.

### Production of monoclonal antibody 6C2

Monoclonal antibody 6C2 was newly prepared using PrP-knockout mice immunized with peptide KTNMKHVAGAAAAG (ovine PrP109–122), conjugated through a cysteine at its C-terminus to Keyhole limpet hemocyanine, using previously described procedures for synthesis and screening [47]. In ELISA and Western blot antibody 6C2 binds respectively to ovine – and bovine recombinant PrP and ovine – and bovine PrP^res ^at the approximate residues HVAGAAA as determined by peptide mapping analysis using an ovine peptide-array.

### Antibody aggregation test

Each reaction contained 500 ng MBP-PrP and monoclonal antibody in PBS containing 0.05% Tween80. For each antibody the inhibitory concentration as well as an excess concentration (max. 25 μg/ml) was tested. The reaction was incubated for 1 hour at room temperature and subsequently centrifuged for 30 minutes at 20,000 × g. Most of the supernatant was transferred to a new tube except approximately 3–5 μl to prevent disturbance of the pellet. The pellet fraction was dissolved in 0.1% SDS by sonification. Both fractions were subjected to methanol precipitation and analyzed by SDS-PAGE (12%, NuPAGE), Western blot and immunodetection using either R521-7 (rabbit anti-PrP serum [48]) and swine anti-rabbit-IgG-peroxidase (PrP detection) or rabbit anti-mouse-IgG-peroxidase (antibody detection). The relative amount of MBP-PrP or antibody band(s) detected as fluorescent signal (f.s.) in Western blot was determined by using the ECF substrate for detection and the Molecular Dynamics ImageQuant software for quantification. Subsequently the mean percentages of MBP-PrP or antibody in the soluble (supernatant) fraction (f.s.^sup^/(f.s.^sup^+f.s.^pel^) were calculated.

### PrP^Sc ^purification and analysis

PrP^Sc ^was isolated from brain tissue of clinically ill scrapie sheep. PrP genotypes were determined by Sanger sequencing of the full PrP-ORF as described before [49]. PrP^Sc ^was purified by ultracentrifugational pelleting from sarcosyl-homogenated brains as described previously [3,50]. The final pellets were sonicated in phosphate-buffered saline containing 1.0% SB 3–14. Yields of PrP^Sc ^were quantified by SDS-PAGE (12% NuPAGE) and Western blotting using antiserum R521-7.

### Reverse detection assay

Confirmed scrapie positive and negative 10% sheep brain homogenates were digested with either proteinase K (PK) or PNGase F and compared to the non-treated samples. A separate aliquot of 10% brain homogenate was treated with 35 μg/ml PK for 1 hour at 37°C. Another aliquot was denatured by adding 1/10 volume denaturing buffer (5% sodium dodecyl sulphate and 10% β-mercaptoethanol in 20 mM Tris-HCl- 150 mM NaCl- 2 mM EDTA [pH 7.5]) for and subsequent heating 10 min. at 96°C. This aliquot was de-glycosylated in the presence of 1000 U PNGase F/ml for at least 36 hours at 37°. Untreated, PK treated – and PNGase F treated brain homogenates samples were analyzed by SDS-PAGE and Western blot. As positive controls His-PrP (positive PrP control) and MBP-PrP (positive detection control) were included. Reverse detection of PrP was accomplished by incubating the Western blot for 1 hour at room temperature with approximately 2 ng/μl MBP-PrP followed by immunodetection using mouse anti-MBP monoclonal antibody and rabbit anti-mouse-IgG-peroxidase (RAMPO). PrP was also detected on Western blot using the PrP-specific monoclonal antibody 9A2 and RAMPO. To determine if detection is PrP specific, a Western blot was carried out with MBP alone instead of MBP-PrP.

## Abbreviations

**TSE**: transmissible spongiform encephalopathy, **PrP**: general denotation for prion protein, **PrP^C^**: host-encoded cellular prion protein (protease sensitive), **PrP^Sc^**: scrapie associated isoform of the prion protein (partially protease resistant), **MBP-PrP**: fusion protein of maltose binding protein linked to the N-terminus of the mature sheep prion protein. **Non-Polar Amino Acids; I**: isoleucine, **V**: valine, **L**: leucine, **F**: phenylalanine, **M**: methionine, **A**: alanine, **G**: glycine, **W**: tryptophane, **P**: proline (ordered from most hydrophobic to most hydrophilic). **Neutral Polar Amino Acids; C**: cysteine, **T**: threonine, **S**: serine, **Y**: tyrosine, **N**: asparagine, **Q**: glutamine (ordered from most hydrophobic to most hydrophilic). **Charged Polar Amino Acids; H**: histidine, **D**: aspartate, **E**: glutamatic acid, **K**: lysine, **R**: arginine (ordered in accordance to increasing hydrophilicity)

## Authors' contributions

AR and AB conceived the study and together with JPML were responsible for study design and coordination. PLJMM was involved in later stage study design and responsible for development and testing of MBP-PrP. JGJ was responsible for the production and testing of monoclonal antibody 6C2. All peptide-arrays were performed at Pepscan B.V. by DPT and all other experiments were performed by AR. JPML and AR were responsible for choice of – and analyses of peptide-arrays. AB is project manager of the NWO-project under which this study was performed. AR and AB were responsible for data-analysis. AR drafted the manuscript and AB and JPML critically read the manuscript before submission. All authors have read and approved the final manuscript.
